# EC2Vec: A Machine Learning Method to Embed Enzyme Commission (EC) Numbers into Vector Representations

**DOI:** 10.1021/acs.jcim.4c02161

**Published:** 2025-02-21

**Authors:** Mengmeng Liu, Xialong Ni, J. Ramanujam, Michal Brylinski

**Affiliations:** † Division of Electrical and Computer Engineering, Louisiana State University, Baton Rouge, Louisiana 70803, United States; ‡ Department of Biological Sciences, Louisiana State University, Baton Rouge, Louisiana 70803, United States; § Center for Computation and Technology, Louisiana State University, Baton Rouge, Louisiana 70803, United States

## Abstract

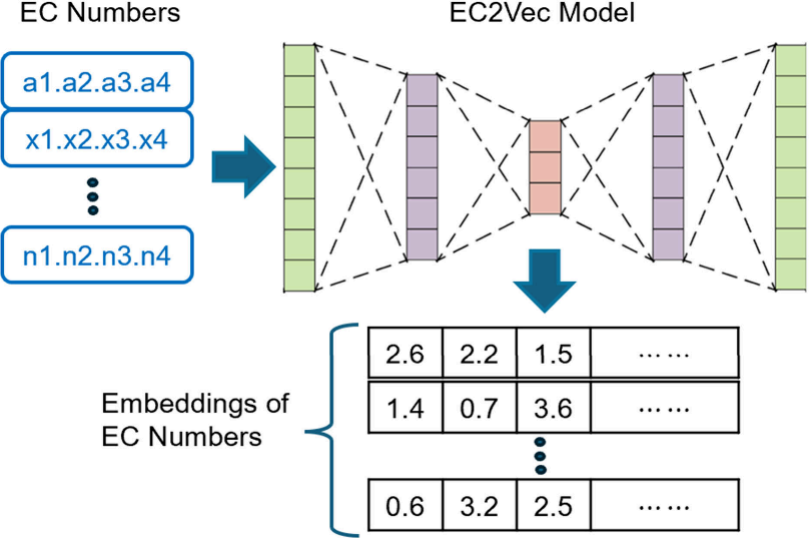

Enzyme commission (EC) numbers play a vital role in classifying enzymes and understanding their functions in enzyme-related research. Although accurate and informative encoding of EC numbers is essential for enhancing the effectiveness of machine learning applications, simple EC encoding approaches suffer from limitations such as false numerical order and high sparsity. To address these issues, we developed EC2Vec, a multimodal autoencoder that preserves the categorical nature of EC numbers and leverages their hierarchical relationships, resulting in more meaningful and informative representations. EC2Vec encodes each digit of the EC number as a categorical token and then processes these embeddings through a 1D convolutional layer to capture their relationships. Comprehensive benchmarking against a large collection of EC numbers indicates that EC2Vec outperforms simple encoding methods. The t-SNE visualization of EC2Vec embeddings revealed distinct clusters corresponding to different enzyme classes, demonstrating that the hierarchical structure of the EC numbers is effectively captured. In downstream machine learning applications, EC2Vec embeddings outperformed other EC encoding methods in the reaction-EC pair classification task, underscoring its robustness and utility for enzyme-related research and bioinformatics applications.

## 1 Introduction

The Enzyme Commission (EC) number system classifies enzymes based on chemical reactions they catalyze. Each EC number consists of four hierarchical levels that describe the enzyme class, subclass, subsubclass, and a specific serial number. This classification system is essential for understanding enzyme functions and facilitating enzyme-related research.^1,2^ Previous works in the domain of enzyme classification and bioinformatics have demonstrated the importance of effective feature encoding to improve model performance.^3−5^ For instance, the application of machine learning to predict enzyme function from protein sequences, structures, and interactions has been explored through various approaches, highlighting the necessity of precise feature representation.^6−8^ Currently, protein sequences are the most commonly used features for encoding enzyme properties. However, sequence-based embeddings have inherent limitations, particularly in cases in which multiple sequences perform the same enzymatic functions, leading to potential redundancy and unreliable results. To overcome these challenges, we developed a novel approach that leverages EC numbers as enzyme features instead of their sequences. Unlike sequence-based embeddings, EC number-based embeddings eliminate redundancy and ambiguity, making them more effective for downstream machine learning tasks. The four-digit EC number system encodes comprehensive information about enzyme functions, including the type of reaction catalyzed and the substrate or mechanism involved, providing highly specific and structured inputs for bioinformatics studies. Despite their potential, research on embedding EC numbers for machine learning and deep learning applications remains limited. This highlights the need for a robust method to accurately translate the functional information encoded in EC numbers into meaningful and actionable embeddings for computational analysis.

A simple method of converting EC numbers to embeddings is to treat each digit in the EC number as a numerical value. This naïve approach implies an inherent order among the digits, which misrepresents their true categorical nature. For example, in an EC number, the digit “2” is not inherently greater than “1”, but rather represents a different category. Another method to encode EC numbers is one-hot encoding, where each digit of the EC number is represented as a one-hot vector. However, this approach leads to high sparsity in encoded vectors. Particularly, the fourth digit of EC numbers can have more than 400 different categories, resulting in vectors where only one bit is set to one, and the remaining hundreds of bits are set to zero. This sparsity can negatively impact the performance and efficiency of machine learning models. Moreover, both methods fail to consider the inherent relationships among the digits of an EC number, leading to the loss of valuable information that is essential for accurately predicting enzyme function and behavior. These limitations in naïve and one-hot encoding methods can result in inaccuracies and suboptimal performance for machine learning models utilizing these embeddings. Consequently, there is a compelling need for an advanced approach to accurately represent the categorical nature of each digit in EC numbers, effectively address issues related to non-numeric characters and sparsity, and account for the relationships among the digits.

To address these challenges, we propose EC2Vec, a multimodal autoencoder that embeds EC numbers in a meaningful and informative way. Our approach treats each digit in the EC number as a categorical token and uses the nn.Embedding method to encode each digit. These digit embeddings are then concatenated and processed through a one-dimensional (1D) convolutional layer to capture the relationships among the digits, effectively learning the hierarchical and functional dependencies within the EC number structure. This method not only preserves the categorical nature of the digits but also leverages their interconnections, resulting in more accurate and informative representations. Our comparative analysis demonstrates that EC2Vec outperforms both naïve numeric embedding and one-hot encoding methods, providing a robust solution for encoding EC numbers in machine learning applications.

## 2 EC2Vec Method

### 2.1 EC Number Representations

**Figure 1:**
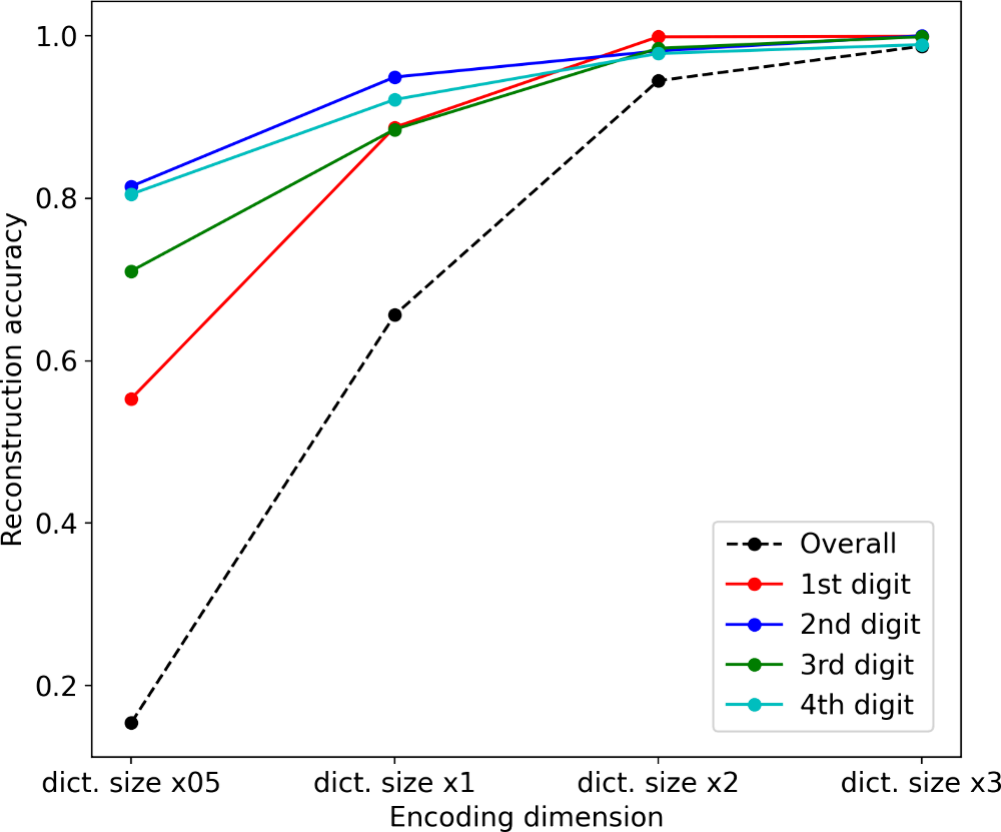
Reconstruction accuracy of EC numbers based on different embedding sizes. The *x*-axis represents the encoding dimensions, with “dict. size ×0.5”, “dict. size ×1”, “dict. size ×2”, and “dict. size ×3” denoting different sizing rules, where “dict. size” refers to the dictionary size of each digit, multiplied by factors of 0.5, 1, 2, and 3. The dictionary sizes for each digit are 8, 28, 34, and 455. For example, “dict. size ×0.5” translates to embedding dimensions of 4, 16, 16, and 256 (after rounding to the closest 2-base number) for the respective digits. The solid lines represent the reconstruction accuracy for each digit, while the dashed line indicates the overall accuracy when all four digits are correctly reconstructed.

In EC2Vec, each digit of the EC number is treated as a categorical token. For example, the EC number for chymotrypsin 3.4.21.1 is tokenized into [“3”, “4”, “21”, “1”]. Each token is converted into an embedding vector using the nn.Embedding method, which treats each digit as a word rather than a number, preserving its categorical nature. The dimension for the embedding vector of each digit is determined by taking twice the number of categories of that digit and rounding to the nearest power of two. Since the number of categories for the first digit is 8, the second 28, the third 34, and the fourth 455, these digits are encoded into 16-, 64-, 64-, and 1024-dimensional vectors, respectively. This selection of embedding dimensions balances the reconstruction accuracy and training efficiency. Embedding dimensions lower than these values lead to worse reconstruction accuracy, while higher dimensions result in longer training times and larger memory consumption without significant gains in accuracy. After each digit is embedded, the resulting vectors are concatenated and passed through a 1D convolutional layer, which captures the relationships among the digits and produces the final EC2Vec embedding. Figure 1 shows the reconstruction accuracy when different embedding sizes are used.

### 2.2 EC2Vec Model Architecture

**Figure 2:**
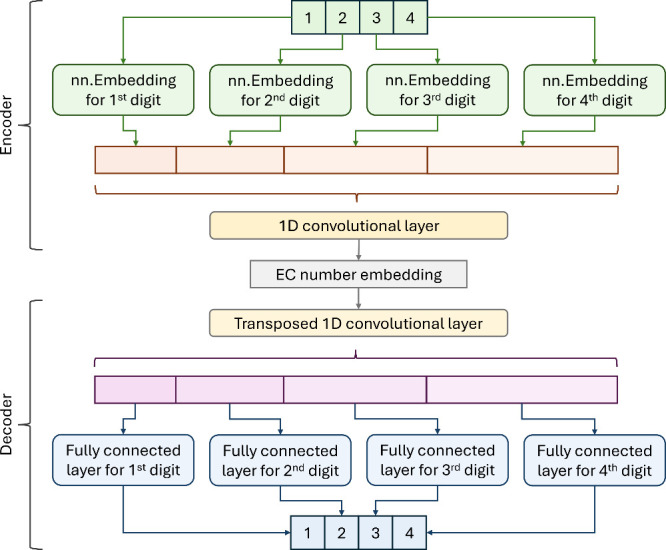
Architecture of the EC2Vec autoencoder. The EC2Vec autoencoder converts an EC number into a vector embedding and reconstructs the original EC number from it. The encoder processes each digit independently using token-based nn.Embedding layers. The embeddings for each digit are then concatenated and passed through a 1D convolutional layer to capture interdigit relationships, producing a cohesive vector representation. The decoder, which mirrors the encoder, uses a transposed 1D convolutional layer and fully connected layers to reconstruct the original EC number. This architecture captures both individual digit characteristics and the hierarchical structure of EC numbers.

The EC2Vec model comprises two main components, the encoder and the decoder, as illustrated in Figure 2. This architecture is designed to embed EC numbers into vector representations and subsequently reconstruct the original EC numbers from these embeddings. The process treats each digit of the EC number as a categorical token, ensuring that the inherent categorical nature of the digits is preserved. The role of the encoder is to transform an EC number into an embedding vector. Each digit of the EC number is processed independently by its respective nn.Embedding layer to obtain a vector representation. The representations of all four digits are concatenated to form a single vector, which is then fed into a 1D convolutional layer. 1D convolutions effectively capture local dependencies in sequential data by applying sliding filters across neighboring embeddings, with respect to their order in the sequence. In the context of EC numbers, this design allows hierarchical relationships, such as enzyme classes influencing subclasses, to naturally emerge from the learned patterns. For example, the convolutional layer can model how enzyme subclasses (second digit) depend on enzyme classes (first digit) or how functional subcategories (third and fourth digits) interact. This mechanism ensures that EC2Vec captures both the individual properties of each digit and its hierarchical interdependence. As a result, the model can effectively capture the relationships among the digits, producing a cohesive embedding that represents the entire EC number. The dimension of the final EC number embedding is determined by the largest dimension among the digit embeddings, which in this case is 1024.

The decoder has a symmetric architecture with respect to the encoder, designed to reconstruct the original EC number from the embedding vector. The embedding first goes through a transposed 1D convolutional layer, mirroring the structure of the encoder convolutional layer and transforming it back into vector representations for each digit. These reconstructed digit vectors are then individually processed through their respective fully connected layers to produce the final reconstructed EC number. The utilization of nn.Embedding to encode the intradigit categorical information and convolutional layers to capture interdigit relationships enhances the ability of the model to generate meaningful and informative embeddings, preserving the categorical relationships and structural hierarchy inherent in the EC number system. The comprehensive architecture and methodology of EC2Vec make it a robust solution for embedding EC numbers, effectively addressing the limitations of the naïve and one-hot encoding methods.

### 2.3 Data to Train EC2Vec

To ensure the comprehensive coverage of existing EC numbers for training the EC2Vec model, and to embed as many EC numbers as possible, we collected EC numbers from EnzyMine,^9^ BRENDA,^10^ Expasy ENZYME,^11^ and UniProt^12^ databases. EnzyMine, a global mining database of enzymatic reactions with annotations in reaction features, provided 7772 unique EC numbers for featured reactions. BRENDA, a well-structured resource offering detailed data on enzymatic reactions, contributed 7831 unique EC numbers across all species. Expasy ENZYME, a library of enzyme nomenclature, provided 8226 unique EC numbers. UniProt, a comprehensive database and a well-known resource for biological researchers, offers data about protein functions and cross-references. To supplement the overall data with valuable information specifically about humans, we collected human enzymes from UniProt, which contained 1766 unique EC numbers. We also included the EC number 0.0.0.0, representing all nonenzyme proteins, in the training data to ensure the completeness of the training examples. After merging all the EC numbers collected from these databases and removing redundancy, we obtained a final data set of 8342 unique EC numbers for training the EC2Vec model.

### 2.4 Managing Category Imbalances in Training

In our training methodology, we tackled the issue of category imbalances in the EC number data set by employing a balanced sampling strategy. The data set exhibits substantial variation in the number of instances across the seven categories, with categories 1 through 7 containing 2590, 2294, 1840, 890, 351, 275, and 101 instances, respectively. To ensure equitable learning across these categories, we assigned inverse frequency weights to each category, enhancing the representation of the underrepresented categories during training. This balanced sampling approach ensures that each category, irrespective of its natural occurrence, receives proportional attention during the model training process, thus mitigating potential biases and enhancing the model generalizability and accuracy across all categories.

## 3 Downstream Machine Learning Applications

### 3.1 Other Approaches

EC2Vec is compared to two simple methods for encoding EC numbers, naïve encoding and one-hot encoding. In naïve encoding, each digit of the EC number is treated as a numerical value, resulting in a raw EC number being converted to a four-dimensional vector. This method misrepresents the categorical nature of the digits and introduces a false numerical order. A model trained on such embeddings will learn the inherent order, which is misleading and can result in incorrect assumptions about the relationships among different EC numbers, ultimately affecting the model performance and accuracy. In one-hot encoding, each digit is represented as a one-hot vector, which effectively preserves the categorical nature of the digits. However, this approach results in significant sparsity, particularly for the fourth digit. The fourth digit can encompass over 400 different categories, leading to highly sparse vectors where only a single bit is set to one and the remaining bits are zeros. This high level of sparsity can negatively impact the efficiency and performance of machine learning models as it introduces challenges in learning meaningful patterns from such sparse representations.

### 3.2 High-Dimensional Data Visualization

**Figure 3:**
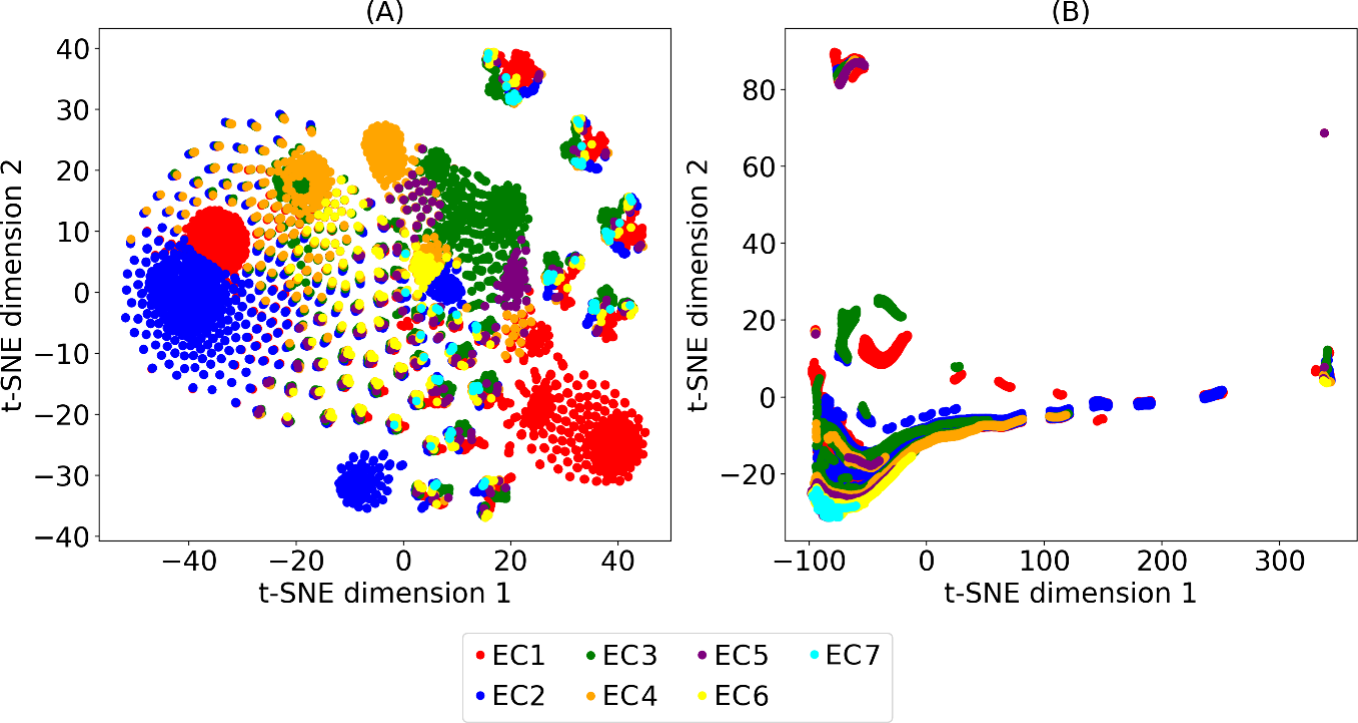
t-SNE visualization of EC2Vec and naïve embeddings of EC numbers. (A) EC2Vec and (B) naïve embeddings in 2D space are colored according to the first EC digit representing main enzyme classes.

The t-Distributed Stochastic Neighbor Embedding (t-SNE)^13^ plot shown in Figure 3(A) visualizes the EC2Vec embeddings of EC numbers, each represented as a 1024-dimensional vector projected into a 2D space. Each point on the plot corresponds to an EC number, with colors indicating the first-digit categories (1 to 7) of the EC numbers. The plot reveals distinct clusters corresponding to these categories, suggesting that the EC2Vec embeddings effectively capture the broad classifications defined by the first digit of the EC number. The clear separation of clusters indicates that the embeddings preserve the categorical distinctions between different enzyme classes, such as oxidoreductases, transferases, and hydrolases. Within each main cluster, there are observable subclusters and variations, highlighting the nuanced differences captured by the embeddings for the subsequent digits of the EC numbers.

The proximity of different clusters may suggest functional similarities or relationships among enzyme classes. For instance, EC1 (oxidoreductases transferring electrons from one molecule to another) and EC2 (transferases transferring group of atoms from one molecule to another) clusters that are close to each other could imply shared characteristics between these enzyme groups. While EC2Vec effectively captures many aspects of enzyme functionality, some finer distinctions between enzyme categories may not be as pronounced due to the inherent limitations of the embedding process and the complexity of enzyme functions. Future work could enhance these representations by incorporating more detailed functional annotations or exploring alternative dimensionality reduction techniques, which may offer different insights into the underlying structure of the data. Additionally, any outliers or points that deviate significantly from the main clusters may represent rare or unique EC numbers that do not conform to typical patterns. To further explore hierarchical relationships, additional t-SNE plots illustrating the clustering of EC numbers based on their subclasses (second digit) are provided as Supporting Information (Figures S1–S7). These plots demonstrate finer-grained clustering within enzyme classes, complementing the analysis of first-digit clustering. Overall, the t-SNE plot demonstrates the capability of the EC2Vec method to produce meaningful and informative embeddings that reflect the hierarchical and functional relationships inherent in the EC number system.

In contrast, the t-SNE plot for the naïve EC number embeddings in Figure 3(B) shows a more dispersed distribution of points. Points corresponding to different categories overlap significantly, indicating that the naïve encoding method, which treats each digit as a numerical value, fails to capture the categorical nature and hierarchical relationships of the EC numbers. The lack of distinct clusters and the scattered nature of the points suggest that this method introduces a false numerical order among the digits, misrepresenting the true relationships between enzyme classes. Additionally, the high sparsity in the plot highlights the inefficacy of this approach in providing meaningful representations of EC numbers. The comparison between these two plots highlights the superiority of the EC2Vec method over the naïve encoding approach. The EC2Vec embeddings produce well-defined and meaningful clusters that reflect the categorical and hierarchical structures of the EC numbers, whereas the naïve encoding method results in a scattered and overlapping distribution that fails to accurately represent the enzyme classifications. This comparison demonstrates that the EC2Vec method offers a more advanced and effective approach to embedding EC numbers.

### 3.3 Prediction of Reaction-EC Pairs

To evaluate the effectiveness of EC2Vec embeddings in downstream machine learning applications, we conducted a classification task using reaction-EC pairs. The goal was to compare EC2Vec embeddings with naïve and one-hot encoding methods. The input to the RF model consisted of two components: compounds (either substrates or products) and EC numbers. Compounds were represented as 300-dimensional vectors generated using Mol2Vec from their Simplified Molecular Input Line Entry System (SMILES) strings.^14^ EC numbers were encoded by using naïve encoding, one-hot encoding, or EC2Vec embeddings. These embeddings were concatenated to form feature vectors, with each row in the resulting matrix representing a reaction-EC pair. For example, a 300-dimensional Mol2Vec compound embedding and a 1024-dimensional EC2Vec embedding were combined into a 1324-dimensional feature vector for each reaction-EC pair. This approach ensured that both the structural properties of the compounds and the functional characteristics of EC numbers were incorporated into the model.

The classification task aimed to distinguish true reaction-EC pairs (positive instances) from false reaction-EC pairs (negative instances). Positive instances were taken directly from the EnzyMine database, which provides enzymatic reactions associated with EC numbers,^15^ and SMILES representations of substrates and products^16^ along with their corresponding EC numbers for each reaction.^9^ Negative instances were created by randomly pairing reactions and EC numbers, then excluding reaction-EC pairs that are present in the positive set. An equal number of positive and negative instances were included in the data set to ensure balanced classes. This procedure resulted in a final data set of 5074 instances.

Table 1 presents the performance comparison of Random Forest (RF)^17^ models utilizing EC embeddings derived from different embedding methods, evaluated using a 5-fold cross-validation protocol. RF based on EC2Vec embeddings consistently demonstrates superior performance across all evaluated metrics, achieving an accuracy of 0.79, the area under the receiver operating characteristic curve (AUC)^18^ of 0.86, and a balanced accuracy of 0.79, significantly outperforming the other methods. The precision (0.81) and recall (0.74) indicate that RF employing EC2Vec embeddings minimizes both false positives and false negatives, resulting in a high F1 score of 0.78 and the lowest False Positive Rate (FPR) of 0.17. The Matthews Correlation Coefficient (MCC)^19^ of 0.57 further supports the robustness of EC2Vec in capturing relevant features and relationships within the EC numbers for the classification task.

**Table 1:** Performance Comparison of Random Forest (RF) Utilizing Different EC Embedding Methods^a^

Method	ACC	AUC	BAC	PPV	TPR	FPR	F1	MCC
EC2Vec	0.785	0.863	0.785	0.811	0.744	0.173	0.776	0.573
Naïve encoding	0.612	0.642	0.612	0.632	0.539	0.314	0.582	0.227
One-hot encoding	0.624	0.666	0.624	0.644	0.555	0.307	0.596	0.251
Baseline	0.541	0.536	0.541	0.547	0.478	0.396	0.510	0.0832

a The performance of RF models using EC embeddings derived from various embedding methods is compared using a 5-fold cross-validation protocol. The results highlight the differences in accuracy and effectiveness of each embedding method. ACC – accuracy, AUC – area under the receiver operating characteristic curve, BAC – balanced accuracy, PPV – precision, TPR – recall, FPR – false positive rate, MCC – Matthews correlation coefficient.

In contrast, RF employing naïve EC embeddings shows relatively poor performance with an accuracy of 0.61, an AUC of 0.64, and a balanced accuracy of 0.61. Its precision (0.63) and recall (0.54) are also lower, leading to an F1 score of 0.58 and a higher FPR of 0.31. The MCC of 0.23 indicates limited effectiveness in capturing useful information for classification. This highlights the limitations of treating EC digits as simple numerical values without accounting for their categorical nature. RF based on a one-hot encoding method performs slightly better than RF based on the naïve EC embedding, with an accuracy of 0.62, an AUC of 0.67, and a balanced accuracy of 0.62. The MCC of 0.25 suggests some improvement over the naïve method but still falls short of the EC2Vec performance. The high sparsity of one-hot vectors, particularly for the fourth digit with over 400 categories, likely contributes to its limited performance. The baseline method employs 1024-dimensional zero vectors as embeddings for EC numbers, thereby omitting any enzyme-related information. Consequently, RF based on this method yields the lowest performance metrics, with an accuracy of 0.54, an AUC of 0.54, a balanced accuracy of 0.54, and an MCC of 0.08, indicating a near-random guessing behavior due to the absence of meaningful EC number representations.

These results clearly demonstrate the superiority of the EC2Vec embedding method in encoding EC numbers for machine learning tasks. By capturing both categorical and hierarchical information, EC2Vec significantly enhances model performance compared to naïve and one-hot encoding methods. The poor performance of the 1024-dimensional zero vector baseline underscores the necessity of incorporating meaningful EC information into the embeddings. The ability of EC2Vec to effectively represent EC numbers translates to better classification accuracy and model robustness, making it a superior choice for enzyme related machine learning applications.

### 3.4 Prediction of Substrate-EC and Product-EC pairs

To further evaluate the effectiveness of embeddings from EC2Vec, we conducted additional classification tasks for substrate-EC and product-EC identification. These tasks enable the assessment of the consistency and effectiveness of EC2Vec embeddings across diverse machine learning contexts, providing deeper insights into how well the embeddings capture and represent the information within EC numbers. Here, a substrate-EC pair indicates that a particular substrate is acted upon by an enzyme corresponding to a specific EC number, while a product-EC pair indicates that a particular product is produced by an enzyme corresponding to a specific EC number. Positive substrate-EC pairs and positive product-EC pairs were obtained from the EnzyMine database. Negative sets for these tasks were created using the same methodology as for the reaction-EC pair negative set; i.e., we randomly paired all substrates or products with EC numbers, excluded pairs present in the positive set, and then randomly sampled an equal number of instances from the resulting set to ensure balanced classes in the classification data set. Following this procedure, the substrate-EC task comprised a total of 4478 instances, while the product-EC task comprised a total of 4,230 instances.

The results presented in Table 2 indicate that the embeddings generated by EC2Vec enable the RF model to achieve high performance across the evaluated tasks, with an AUC of 0.84 for substrate-EC pair identification and 0.81 for product-EC pair identification. The consistently strong performance of the RF classifier using EC2Vec embeddings across diverse classification tasks highlights the effectiveness of EC2Vec in capturing the intrinsic properties of EC numbers. This demonstrates the utility of EC2Vec as a valuable tool for enzyme function analysis and other related bioinformatics applications.

**Table 2:** Performance of RF Models Utilizing EC2Vec Embeddings on Substrate-EC and Product-EC Identification Tasks^a^

Task	ACC	AUC	BAC	PPV	TPR	FPR	F1	MCC
Substrate-EC	0.775	0.843	0.775	0.864	0.653	0.103	0.744	0.567
Product-EC	0.734	0.811	0.733	0.813	0.605	0.138	0.694	0.483

a Classification tasks were conducted using RF classifiers to evaluate the effectiveness of EC2Vec embeddings in determining the presence of substrate-EC or product-EC pairs. These tasks were performed using a 5-fold cross-validation protocol to ensure robust evaluation. ACC – accuracy, AUC – area under the receiver operating characteristic curve, BAC – balanced accuracy, PPV – precision, TPR – recall, FPR – false positive rate, MCC – Matthews correlation coefficient

## 4 Conclusions

This study highlights the substantial advantages of the EC2Vec embedding method over simple encoding techniques for representing EC numbers. Our comparative analysis demonstrates that EC2Vec significantly enhances the performance of machine learning models by effectively capturing the hierarchical and categorical nature of EC numbers. Naïve encoding treats each digit as a numerical value, imposing a false ordinal relationship that does not reflect the true categorical distinction among the EC digits. This misrepresentation can lead to inaccurate assumptions about enzyme functions, adversely affecting the model performance. One-hot encoding, while preserving categorical distinctions, suffers from extreme sparsity, particularly for the fourth digit with its large number of categories. This sparsity complicates the learning process, leading to suboptimal performance in machine learning tasks. EC2Vec effectively addresses these issues by treating each digit as a categorical token and generating embeddings that capture both individual and relational properties. This approach preserves the hierarchical structure of EC numbers, leading to more meaningful and informative representations.

The success of EC2Vec across various classification tasks highlights its potential as a valuable tool for enzyme research and bioinformatics applications. By providing a precise and informative representation of EC numbers, EC2Vec enhances enzyme function prediction, specificity analysis, and understanding of enzymatic reactions. For multifunctional enzymes, EC2Vec can be adapted by incorporating additional labels, effectively capturing the unique enzymatic functions associated with multiple EC numbers. This approach focuses on functional distinctions rather than sequence variations, making it particularly effective for studying enzymes with diverse activities. The ability of EC2Vec to encode both categorical and hierarchical information positions it as a robust framework for advancing computational models in enzyme research. Future refinements could include integrating additional contextual information or enhancing the model architecture. Expanding its evaluation to a wider range of enzyme-related tasks and data sets would further assess its generalizability and applicability across diverse bioinformatics challenges. EC2Vec can help fill missing links in metabolic pathways, annotate enzymes where sequence or structural data are incomplete, and explore microbial metabolism. For instance, we are currently using EC2Vec embeddings to predict whether specific metabolites are consumed or produced by combinations of bacterial enzymes in the human gut. Preliminary results indicate that this approach achieves an initial accuracy of approximately 0.7 when it is applied to microbial enzyme combinations. These findings highlight the potential of EC2Vec to unravel interactions between the gut microbiome and human metabolism, offering valuable insights into the microbial contributions to health and disease.

In conclusion, EC2Vec represents a significant advancement in encoding EC numbers for machine learning applications. Its ability to capture the hierarchical and categorical nature of EC numbers translates into improved model performance and offers a robust solution for enzyme-related research and bioinformatics applications.

## Data Availability

EC2Vec is available at https://github.com/MengLiu90/EC2Vec under a GPL-3.0 license.

## Supporting Information Available

t-SNE plots of EC number subclasses for 7 enzyme classes (PDF)
