# Supplementary material for: EC2Vec: A Machine Learning Method to Embed Enzyme Commission (EC) Numbers into Vector Representations
Source: J Chem Inf Model. 2025 Feb 21;65(5):2173–9. doi: 10.1021/acs.jcim.4c02161 (PMC11898066; doi:10.1021/acs.jcim.4c02161)
Supplement: Supplementary file 1 — ci4c02161_si_001.pdf [file ci4c02161_si_001.pdf]

Supporting information for EC2Vec: a machine learning tool to embed enzyme commission (EC) numbers into vector representations

Mengmeng Liu[1], Xialong Ni[2], J. Ramanujam[1,3], and Michal Brylinski[2,3*]

[1] Division of Electrical and Computer Engineering, Louisiana State University, Baton Rouge, LA, 70803, USA

[2] Department of Biological Sciences, Louisiana State University, Baton Rouge, LA 70803, USA

[3] Center for Computation and Technology, Louisiana State University, Baton Rouge, LA 70803, USA

This supplementary material provides additional t-SNE visualizations to explore the hierarchical relationships between enzyme classes and their corresponding subclasses (second digit) in EC numbers. Figures S1-S7 illustrate the subclass distribution for each of the seven enzyme classes, complementing the analysis presented in the main manuscript. These t-SNE visualizations were generated using scikit-learn with a perplexity of 30 and learning rate of 200. EC numbers were encoded using the EC2Vec embeddings as described in the main text.

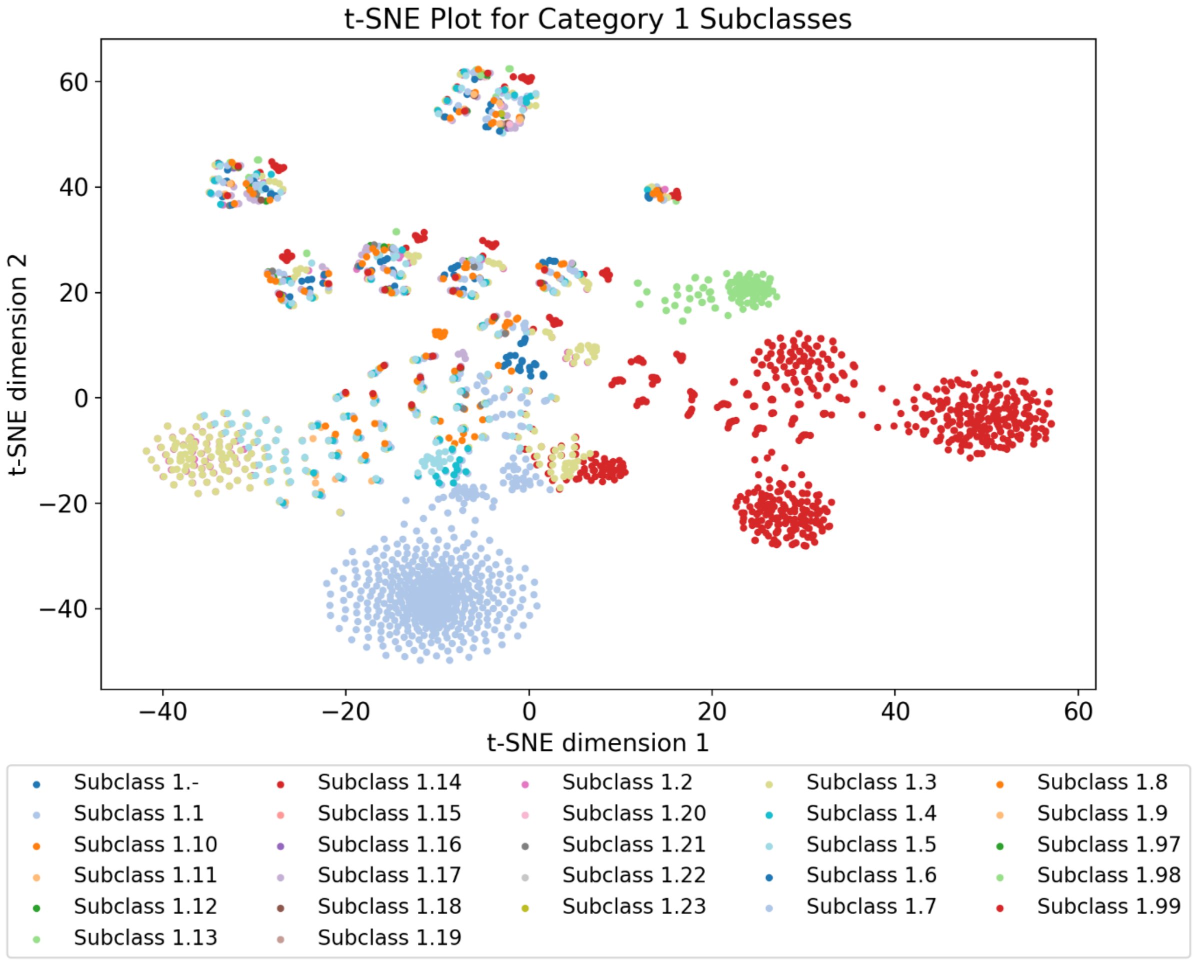

Figure S1. t-SNE plot of EC number subclasses for enzyme class 1 (oxidoreductases). Each point represents an EC number, color-coded by subclass (second digit). The clustering highlights relationships between subclasses within this enzyme class.

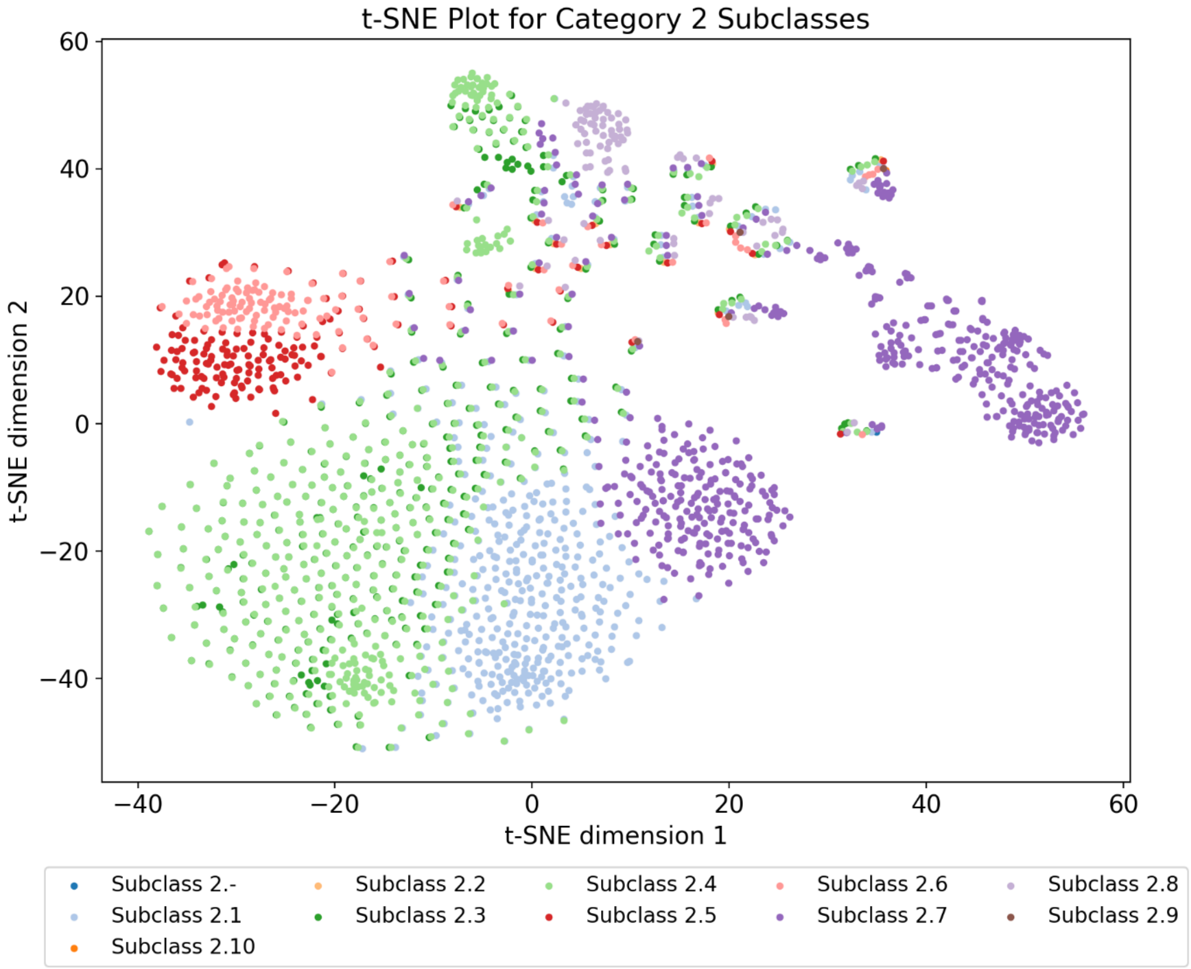

Figure S2. t-SNE plot of EC number subclasses for enzyme class 2 (transferases). Each point represents an EC number, color-coded by subclass (second digit).

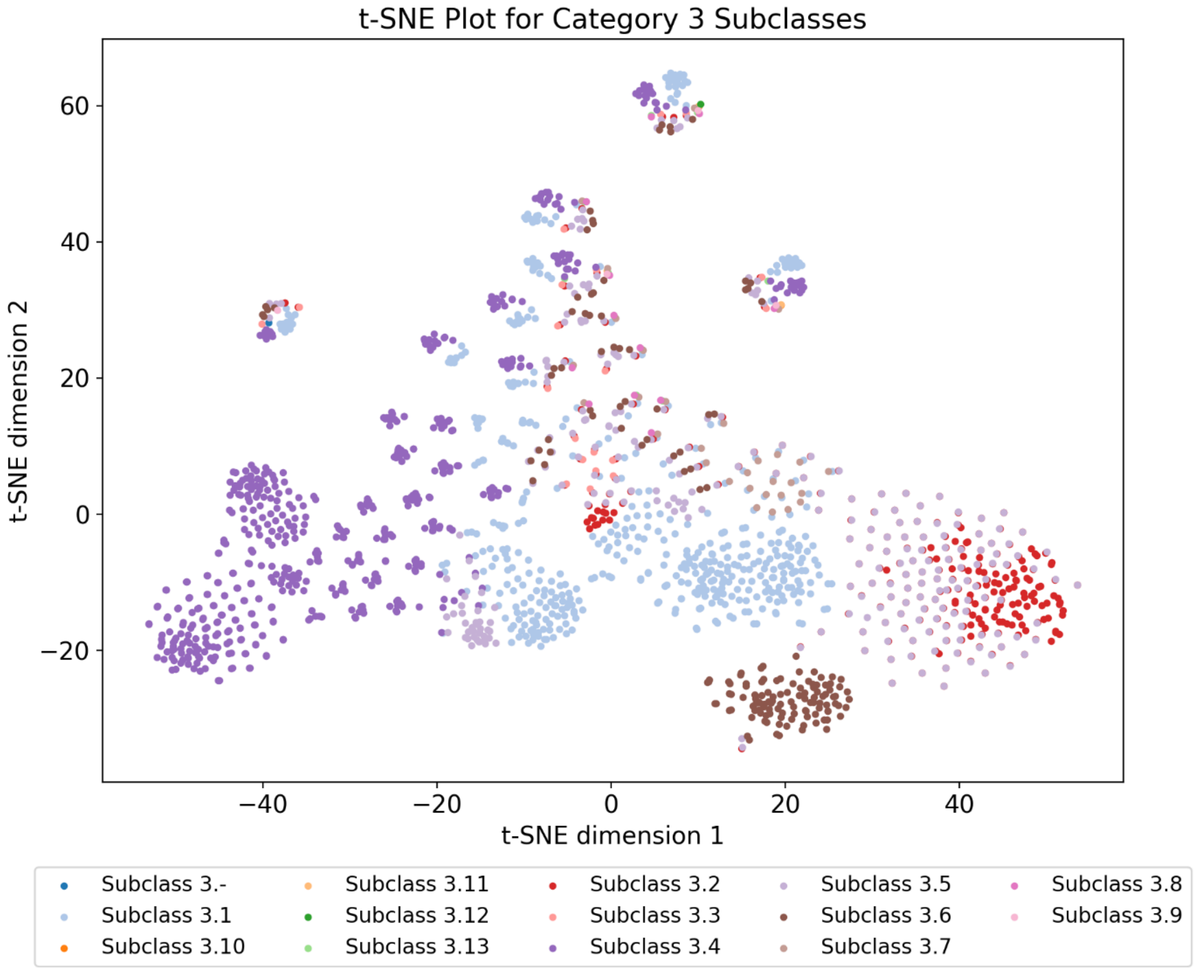

Figure S3. t-SNE plot of EC number subclasses for enzyme class 3 (hydrolases).

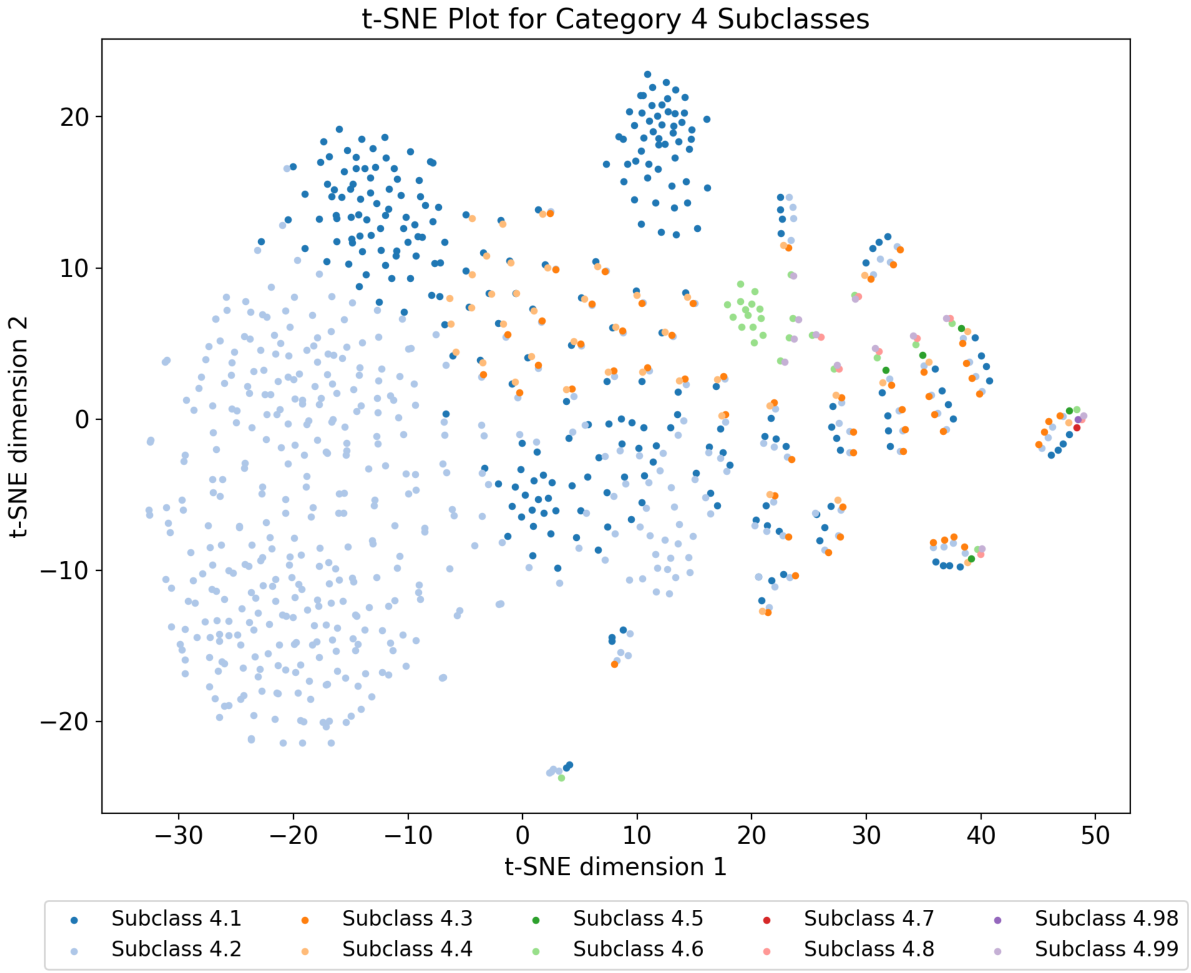

Figure S4. t-SNE plot of EC number subclasses for enzyme class 4 (lyases).

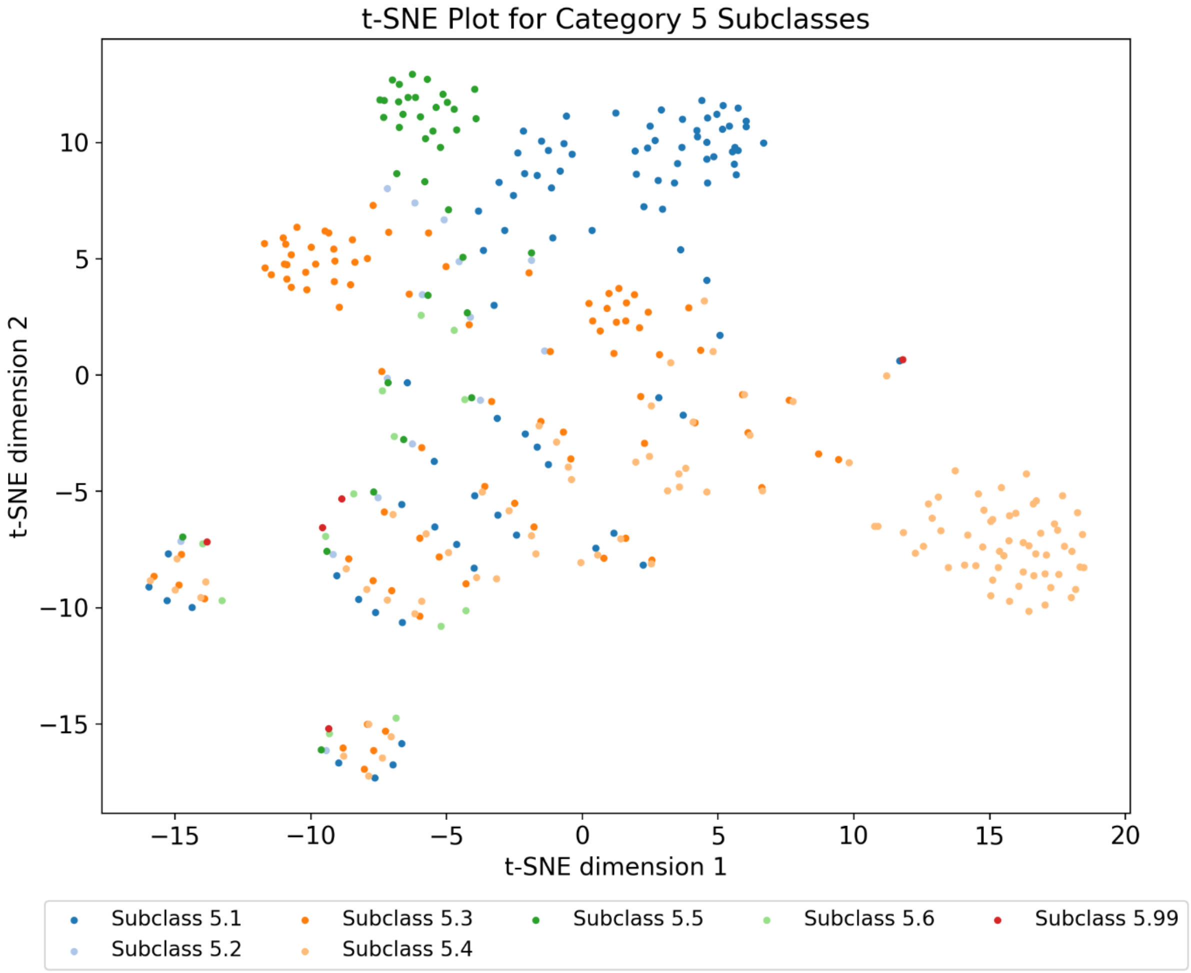

Figure S5. t-SNE plot of EC number subclasses for enzyme class 5 (isomerases).

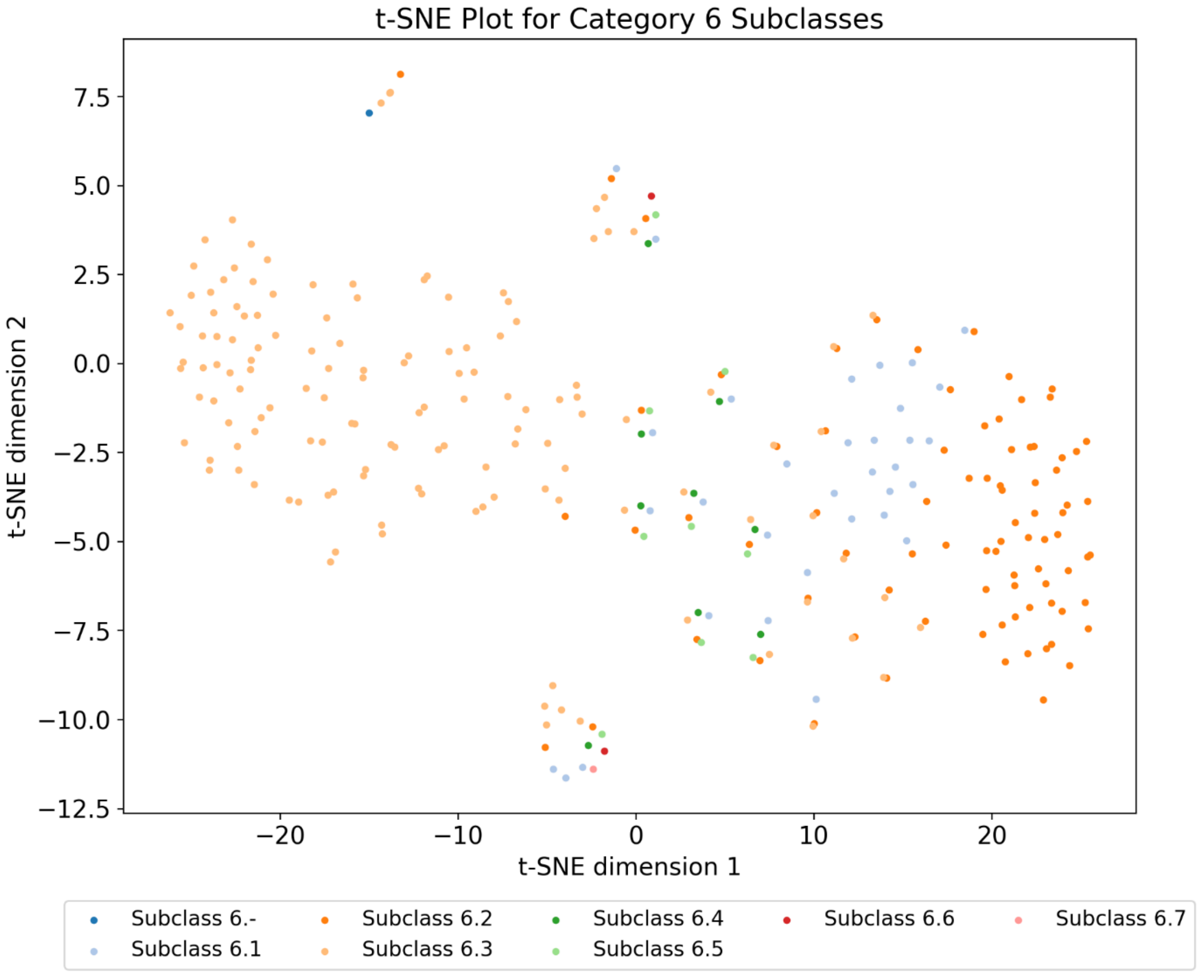

Figure S6. t-SNE plot of EC number subclasses for enzyme class 6 (ligases).

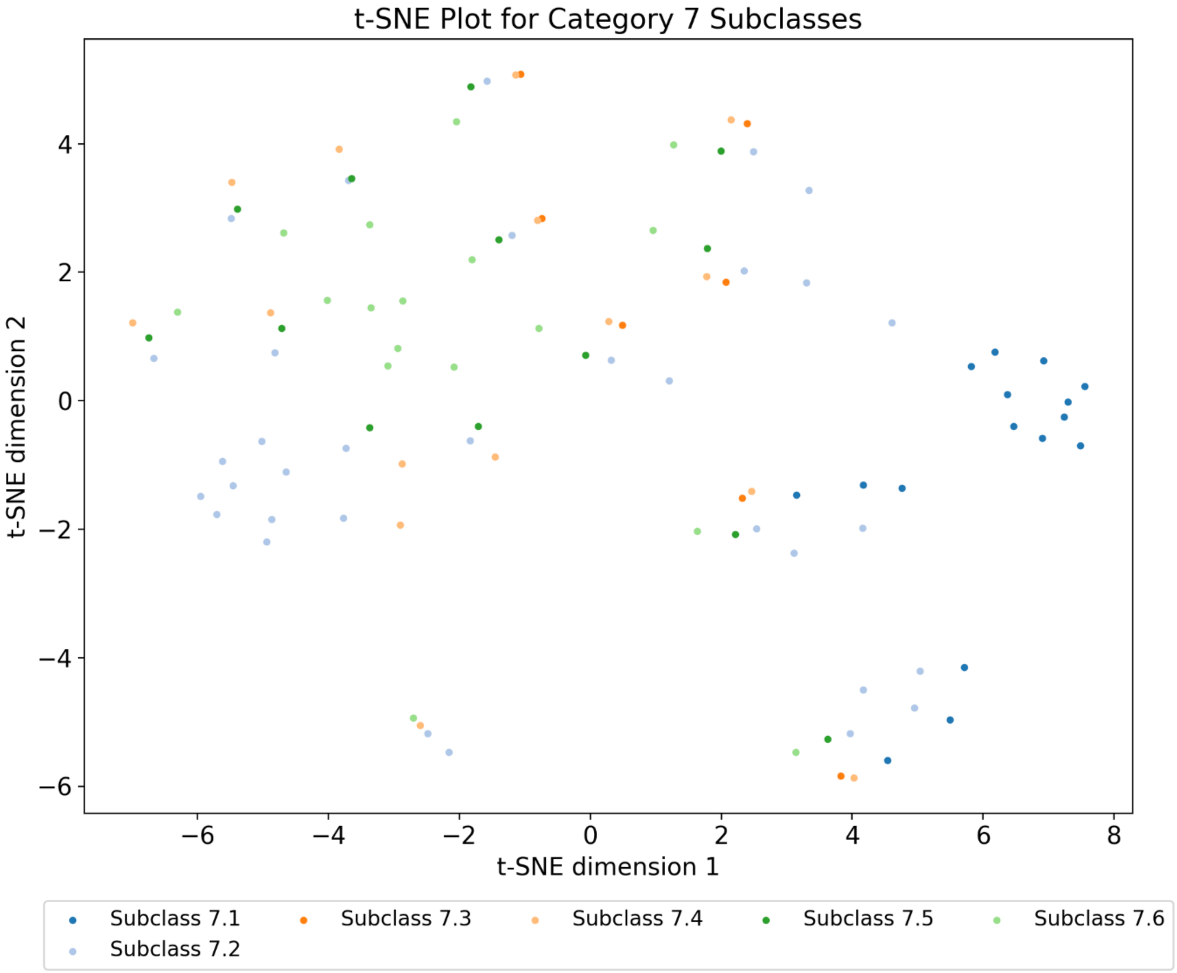

Figure S7. t-SNE plot of EC number subclasses for enzyme class 7 (translocases).
